# Supplementary material for: Ion counting demonstrates a high electrostatic field generated by the nucleosome
Source: eLife. 2019 Jun 11;8:e44993. doi: 10.7554/eLife.44993 (PMC6584128; doi:10.7554/eLife.44993)
Supplement: Figure 3—source data 1. [file elife-44993-fig3-data1.pdf]

Figure 3 - Source Data 1: Experimentally determined excess number (N_i), the β_+ coefficient (the faction of associated cations), and the β_- coefficient (the faction of excluded anions)) for 10 mM NaBr around 147 bp DNA

	free dsDNA			
	N_{Na^+}	N_{Br^-}	total (experimental)	$q_{molecule}$ (theory)
	247± 4.9	-45 ± 2.9	292 ± 4.9	-292
β_+	0.846 ± 0.019			
β_-	0.157 ± 0.008			
