# Supplementary material for: Ion counting demonstrates a high electrostatic field generated by the nucleosome
Source: eLife. 2019 Jun 11;8:e44993. doi: 10.7554/eLife.44993 (PMC6584128; doi:10.7554/eLife.44993)
Supplement: Figure 3—source data 2. [file elife-44993-fig3-data2.pdf]

Figure 3 - Source Data 2: Experimentally determined excess number (N_i), the β_+ coefficient (the faction of associated cations), and the β_- coefficient (the faction of excluded anions) for 10 mM NaBr around canonical nucleosome

	**canonical nucleosome**			
	N_{Na^+}	N_{Br^-}	total (experimental)	$q_{molecule}$ (theory)*
	119 ± 1.7	-24 ± 2.6	144 ± 1.7	-144
β_+	0.826 ± 0.020			
β_-	0.166 ± 0.015			

* overall charge of the 147bp DNA is -292e and the overall charge of the histone core based on amino acid composition is +148e (Table S8)
