# Supplementary material for: Ion counting demonstrates a high electrostatic field generated by the nucleosome
Source: eLife. 2019 Jun 11;8:e44993. doi: 10.7554/eLife.44993 (PMC6584128; doi:10.7554/eLife.44993)
Supplement: Figure 3—source data 3. [file elife-44993-fig3-data3.pdf]

Figure 3 - Source Data 3: Experimentally determined excess number (N_i), the β_+ coefficient (the faction of associated cations), and the β_- coefficient (the faction of excluded anions) for 10 mM NaBr around H3 tailless nucleosome

	**H3 tailless nucleosome**			
	N_{Na^+}	N_{Br^-}	total (experimental)	$q_{molecule}$ (theory)
	140 ± 4.2	-18 ± 4.0	160 ± 3.1	-160
β_+	0.875 ± 0.025			
β_-	0.112 ± 0.020			
