# Supplementary material for: Ion counting demonstrates a high electrostatic field generated by the nucleosome
Source: eLife. 2019 Jun 11;8:e44993. doi: 10.7554/eLife.44993 (PMC6584128; doi:10.7554/eLife.44993)
Supplement: Figure 3—source data 4. [file elife-44993-fig3-data4.pdf]

**Figure 3 - Source Data 4: Experimentally determined excess number ($N_i$) and the $\beta_+$ for 25 mM NaBr and 2.5 mM $MgBr_2$ around 24 bp DNA, 147 bp DNA and H3 tailless nucleosome**

| | 24 bp DNA | | | | 147 bp DNA | | | | H3 tailless nucleosome | | | |
|---|---|---|---|---|---|---|---|---|---|---|---|---|
| | $N_{Na^+}$ | $N_{Mg^{2+}}$ | $N_{Br^-}$ | total | $N_{Na^+}$ | $N_{Mg^{2+}}$ | $N_{Br^-}$ | total | $N_{Na^+}$ | $N_{Mg^{2+}}$ | $N_{Br^-}$ | total |
| | 14.0 ± 1.0 | 13.5± 0.5 | -4.5 ±1.0 | 45.0 ± 1.0 | 81.0 ± 2.0 | 95.0 ± 1.0 | -25.2 ± 1.1 | 296 ± 2.3 | 21.0 ± 1.5 | 58.0 ± 1.0 | -21.0 ± 1.0 | 157.0 ± 1.8 |
| $Mg^{2+}$:$Na^+$ | 1:0.97 | | | | 1.16:1 | | | | 2.76:1 | | | |
