# Supplementary material for: Ion counting demonstrates a high electrostatic field generated by the nucleosome
Source: eLife. 2019 Jun 11;8:e44993. doi: 10.7554/eLife.44993 (PMC6584128; doi:10.7554/eLife.44993)
Supplement: Figure 3—source data 5. [file elife-44993-fig3-data5.pdf]

Figure 3 - Source Data 5: PB calculation of excess number (N_i) and the β_- coefficient (the faction of associated anions), and the β_+ coefficient (the faction of excluded cation) for 40 mM NaBr around histone octamer

	**histone octamer**		
	N_{Na^+}	N_{Br^-}	Total*
	-24	125	-149
β_+	0.161		
β_-	0.838		

* Charges were assigned using the PDB2PQR routine with the Amber parameter, at pH = 7.5
