# Supplementary material for: Ion counting demonstrates a high electrostatic field generated by the nucleosome
Source: eLife. 2019 Jun 11;8:e44993. doi: 10.7554/eLife.44993 (PMC6584128; doi:10.7554/eLife.44993)
Supplement: Figure 3—source data 6. [file elife-44993-fig3-data6.pdf]

Figure 3 - Source Data 6: Experimentally determined excess number (N_i) and the β_- coefficient (the faction of associated anions) and the β_+ coefficient (the faction of excluded cation) for 40 mM NaBr around histones H3, H4 and $(H3\cdot H4)_2$ tetramer

	H3				H4				$(H3\cdot H4)_2$ tetramer			
	N_{Na^+}	N_{Br^-}	total	q_{H3}	N_{Na^+}	N_{Br^-}	total	q_{H4}	N_{Na^+}	N_{Br^-}	total	q_{com}
	-4.6 ± 1.0	14.0 ± 1.5	-18.6 ± 1.8	+20	-4.9 ± 1.0	13.3 ± 0.5	-18.2 ± 1.1	+18	-20.3 ± 0.5	57.3 ± 1.7	-77.6 ± 1.8	+76
β_-	0.74 ± 0.050				0.71 ± 0.05				0.74 ± 0.006			
β_+	0.25 ± 0.05				0.27 ± 0.05				0.26 ± 0.012			
