# Supplementary material for: Ion counting demonstrates a high electrostatic field generated by the nucleosome
Source: eLife. 2019 Jun 11;8:e44993. doi: 10.7554/eLife.44993 (PMC6584128; doi:10.7554/eLife.44993)
Supplement: Figure 3—source data 7. [file elife-44993-fig3-data7.pdf]

Figure 3 - Source Data 7: Experimentally determined excess number (N_i) and the β_- coefficient (the faction of associated anions) and the β_+ coefficient (the faction of excluded cation) for 40 mM NaBr around histones H2A, H2B and (H2A·H2B) dimer

	H2A				H2B				(H2A·H2B) dimer			
	N_{Na^+}	N_{Br^-}	total	q_{H2A}	N_{Na^+}	N_{Br^-}	total	q_{H2B}	N_{Na^+}	N_{Br^-}	total	q_{com}
	-4.7 ± 1.5	10.2 ± 1.0	-15.0 ± 1.8	+17	-5.0 ± 1.0	13.6 ± 1.0	-18.6 ± 1.0	+19	-8.5 ± 2.0	26.5 ± 1.8	-35.0 ± 2.2	+36
β_-	0.69 ± 0.060				0.73 ± 0.04				0.75 ± 0.02			
β_+	0.32 ± 0.1				0.27 ± 0.05				0.24 ± 0.05			
