# Supplementary material for: Ion counting demonstrates a high electrostatic field generated by the nucleosome
Source: eLife. 2019 Jun 11;8:e44993. doi: 10.7554/eLife.44993 (PMC6584128; doi:10.7554/eLife.44993)
Supplement: Transparent reporting form [file elife-44993-transrepform.docx]

***eLife’s* transparent reporting form**

We encourage authors to provide detailed information *within their submission* to facilitate the interpretation and replication of experiments. Authors can upload supporting documentation to indicate the use of appropriate reporting guidelines for health-related research (see [EQUATOR Network](http://www.equator-network.org/%20)), life science research (see the [BioSharing Information Resource](https://biosharing.org/)), or the [ARRIVE guidelines](http://www.plosbiology.org/article/info:doi/10.1371/journal.pbio.1000412) for reporting work involving animal research. Where applicable, authors should refer to any relevant reporting standards documents in this form.

If you have any questions, please consult our Journal Policies and/or contact us: [editorial@elifesciences.org](mailto:editorial@elifesciences.org).

**Sample-size estimation**

- You should state whether an appropriate sample size was computed when the study was being designed
- You should state the statistical method of sample size computation and any required assumptions
- If no explicit power analysis was used, you should describe how you decided what sample (replicate) size (number) to use

Please outline where this information can be found within the submission (e.g., sections or figure legends), or explain why this information doesn’t apply to your submission:

Sample sizes were not computed in advance.

**Replicates**

- You should report how often each experiment was performed
- You should include a definition of biological versus technical replication
- The data obtained should be provided and sufficient information should be provided to indicate the number of independent biological and/or technical replicates
- If you encountered any outliers, you should describe how these were handled
- Criteria for exclusion/inclusion of data should be clearly stated
- High-throughput sequence data should be uploaded before submission, with a private link for reviewers provided (these are available from both GEO and ArrayExpress)

Please outline where this information can be found within the submission (e.g., sections or figure legends), or explain why this information doesn’t apply to your submission:

Ion counting results reported in Figure 3A-3F were collected from 2-3 independent experiments (i.e. ‘biological’ replicate). An independent experiment is defined as an experiment carried out on a different day, using freshly prepared biological samples such as 147bp DNA, canonical and H3 tailless nucleosomes, and histone proteins. For each biological replicate, 2 sample (or ‘technical’) replicates were prepared. Each technical replicate was analyzed 3-times by inductively coupled plasma mass spectrometry (ICP MS) using a XSERIES 2 ICP-MS (Thermo Scientific, USA) to measure the concentration of specific atoms (i.e. ‘measurement’ replicates). Measurements replicates (i.e. instrument precision) varied less than 3%.

The reported errors are the standard deviations of all biological and technical replicates for a given sample; no data points were excluded. A description of the sample preparation and measurements is provided in the Material and Methods section of the manuscript.

**Statistical reporting**

- Statistical analysis methods should be described and justified
- Raw data should be presented in figures whenever informative to do so (typically when N per group is less than 10)
- For each experiment, you should identify the statistical tests used, exact values of N, definitions of center, methods of multiple test correction, and dispersion and precision measures (e.g., mean, median, SD, SEM, confidence intervals; and, for the major substantive results, a measure of effect size (e.g., Pearson's r, Cohen's d)
- Report exact p-values wherever possible alongside the summary statistics and 95% confidence intervals. These should be reported for all key questions and not only when the p-value is less than 0.05.

Please outline where this information can be found within the submission (e.g., sections or figure legends), or explain why this information doesn’t apply to your submission:

The bar graphs in Figure 3A-3F show mean values and the error bars represent standard deviations. In addition, the mean values and standard deviations for all experiments are summarized in tables available in the supporting information (Source Data 1-7). As described above, mean values and standard deviations were derived based on all biological and technical replicates of a given sample.

To compare the polyelectrolyte character of canonical and H3-tailless nucleosomes, mean beta-values derived from ion counting experiments (see section ‘Strategy to measure nucleosome electrostatics’ in the main text) were analyzed using a two-sample t-test. This revealed that the mean beta-values of canonical (N = 3; mean = 0.825; SD = 0.02) and H3-tailless nucleosomes (N = 3; mean = 0.88; SD = 0.02) were significantly different, with p = 0.028. Statistical analysis was carried out using OriginPro2017 (OriginLab, Northampton, USA).

(For large datasets, or papers with a very large number of statistical tests, you may upload a single table file with tests, Ns, etc., with reference to sections in the manuscript.)

**Group allocation**

- Indicate how samples were allocated into experimental groups (in the case of clinical studies, please specify allocation to treatment method); if randomization was used, please also state if restricted randomization was applied
- Indicate if masking was used during group allocation, data collection and/or data analysis

Please outline where this information can be found within the submission (e.g., sections or figure legends), or explain why this information doesn’t apply to your submission:

N/A

**Additional data files (“source data”)**

- We encourage you to upload relevant additional data files, such as numerical data that are represented as a graph in a figure, or as a summary table
- Where provided, these should be in the most useful format, and they can be uploaded as “Source data” files linked to a main figure or table
- Include model definition files including the full list of parameters used
- Include code used for data analysis (e.g., R, MatLab)
- Avoid stating that data files are “available upon request”

Please indicate the figures or tables for which source data files have been provided:

N/A
